# Improving the Detection of Cholangiocarcinoma: In vitro MRI-Based Study Using Local Coils and T2 Mapping

**DOI:** 10.2147/HMER.S232392

**Published:** 2020-03-24

**Authors:** Narong Khuntikeo, Attapol Titapun, Nittaya Chamadol, Wuttisak Boonphongsathien, Prakasit Sa-Ngiamwibool, Simon D Taylor-Robinson, Christopher A Wadsworth, Shuo Zhang, Evdokia M Kardoulaki, Ian R Young, Richard R A Syms

**Affiliations:** 1 Department of Surgery, Faculty of Medicine, Khon Kaen University, Khon Kaen 40002, Thailand; 2 Department of Radiology, Faculty of Medicine, Khon Kaen University, Khon Kaen 40002, Thailand; 3 Department of Pathology, Faculty of Medicine, Khon Kaen University, Khon Kaen 40002, Thailand; 4 Division of Surgery and Cancer, Imperial College London, Liver Unit, St. Mary’s Hospital, London W2 1NY, UK; 5 Philips Healthcare Germany, Health Systems, Clinical Science, Hamburg 22335, Germany; 6 EEE Department, Imperial College, London SW7 2AZ, UK

**Keywords:** cholangiocarcinoma, T2 mapping, internal coil

## Abstract

**Aim:**

Cholangiocarcinoma is endemic in southeast Asia, generally developing from liver fluke infestation. However, diagnostic imaging of early-stage disease is challenging. The aim of this work is to investigate relaxometry (specifically, T2 mapping) as a method of exploiting the higher signal-to-noise ratio (SNR) of internal coils for improved reception of magnetic resonance signals, despite their non-uniform sensitivity.

**Methods:**

Ex vivo T2 mapping was carried out at 3T on fixed resection specimens from Thai cholangiocarcinoma patients using an mGRASE sequence and an endoscope coil based on a thin-film magneto-inductive waveguide and designed ultimately for internal use.

**Results:**

Disease-induced changes including granulomatous inflammation, intraepithelial neoplasia and intraductal tumours were correlated with histopathology, and relaxation data were compared with mono- and bi-exponential models of T2 relaxation. An approximately 10-fold local advantage in SNR compared to a 16-element torso coil was demonstrated using the endoscope coil, and improved tissue differentiation was obtained without contrast agents.

**Conclusion:**

The performance advantage above follows directly from the inverse relation between the component of the standard deviation of T2 due to thermal noise and the SNR, and offers an effective method of exploiting the SNR advantage of internal coils. No correction is required, avoiding the need for tracking, relaxing constraints on coil and slice orientation and providing rapid visualization.

## Introduction

Cholangiocarcinoma (CCA) is an adenocarcinoma of the biliary ductal system.^1^ It is rare in the West (although incidence is rising^2^,^3^) but endemic in southeast Asian countries, where the cause is generally infestation with liver fluke following consumption of uncooked river fish.^4^ Cysts hatch in the duodenum, migrate into the bile duct and mature in the extrahepatic bile ducts. Adult activity causes damage to the biliary epithelium. Eggs become trapped in periductal tissue and induce granulomatous inflammation. Toxic excretions cause chronic irritation and epithelial hyperplasia that can eventually lead to oxidative DNA damage and malignant transformation.^5^ Despite education programs, an estimated 9.4% of the Thai population (around 6 million people) is affected with fluke. Khon Kaen University Hospital is the centre for treatment in Isaan province, where CCA is responsible for 25,000 deaths per year.^6^

Precursors of CCA include egg-induced granulomatous inflammation, periductal fibrosis and intraepithelial neoplasia. Tumours may be intrahepatic and extrahepatic^7^ and are classified into mass forming, periductal infiltrating and intraductal types.^8^,^9^ There is significant histological variation due to cholangiocyte diversity^10^ and updated classifications have been proposed.^11^ CCA is a silent disease, with few early symptoms (lethargy, weight loss, abdominal pain, itching) although obstructive jaundice may occur as intraductal tumours develop. Biomarkers still lack sufficient sensitivity and specificity for screening.^12^ Ultrasound has been successful in identifying mass-forming lesions and periductal fibrosis.^13^ However, periductal and intraductal tumours are more difficult to detect, so patients typically present in their early 60s when disease is advanced. CCA is resistant to radiotherapy and chemotherapy,^14^ and surgery offers the main possibility of a cure; however, it is difficult to achieve sufficient resection margin and the 5-year survival rate is low.^15^

Diagnostic imaging is performed using computed tomography or magnetic resonance imaging (MRI); here we focus on the latter. Affected ducts appear with thickened, irregular, asymmetric or indistinct outer walls, and CCA is often inferred from dilation or constriction.^16^ Due to neovascularization, inflammatory lesions and intraductal tumours have different enhancement patterns in the hepatic arterial, portal venous and delayed phases of dynamic contrast imaging.^17^ In advanced cases, imaging appearance and correlation with histopathology are well established.^18^ However, not all tumours present with a classical appearance, especially early-stage cases that might be detected by ultrasound examination.^19^ Technical advances and multimodal imaging are therefore needed to differentiate inflammatory from malignant lesions.^20^

This paper considers the use of internal detection coils to improve tissue differentiation, by virtue of the increased local signal-to-noise ratio (SNR) obtained from a reduced field-of-view (FOV) for body noise.^21^ Many such coils have been demonstrated, with introduction via a catheter^22^,^23^ or an endoscope.^24^ However, all have spatially variable signal reception. The need for image correction has then often outweighed any advantage, as have safety considerations.^25^ Most have been intended for vascular or gastrointestinal imaging, and biliary imaging has received little attention.^26^ We have developed a prototype duodenoscope with a signal-receiving sheath along its shaft for this purpose.^27^ Here we aim to clarify any potential advantage of locally increased SNR, using relaxometry to mitigate non-uniform reception.

Relaxometry uses multiple measurements to estimate relaxation time constants, and therefore depends less on absolute signal level.^28^ Here we consider the spin-spin time constant, T2. Due to local differences in proton environment, relaxation processes may be multi-exponential,^29^ and time, temperature and fixation all alter relaxation ex vivo.^30^,^31^ Measurements are also affected by diffusion^32^ and field strength.^33^ The reproducibility of estimated T2 values was therefore initially low,^34^ even with standard test objects,^35^ and their diagnostic significance was correspondingly limited.^36^ However, sequences have now been developed to minimize diffusion sensitivity^37^–^39^ and increase speed,^40^,^41^ and many tissue types have been characterized.^42^

Time constants are estimated by non-linear least squares matching to a model. Fitting may be highly inaccurate at low SNR, leading to a bias in estimated value.^43^,^44^ However, bias largely disappears at high SNR. The residual effect of noise is to convert time constants into distributions of values, which limit differentiation of tissues with similar T2. An inverse relation between SNR and the standard deviation σ_T2N_ of T2 due to noise is known,^45^,^46^ suggesting that differentiation will increase at higher SNR. Matching to bi-exponential models will also be improved, since this requires inherently higher SNR.^47^,^48^

No relaxation experiments appear to have been performed with internal coils, or on diseased biliary ductal issue. The aim of this work was to investigate both aspects by performing an ex vivo investigation of CCA resection specimens employing an endoscope coil for signal reception, using comparison with an external coil to determine whether a local SNR advantage can improve T2 mapping sufficiently to increase tissue differentiation in the absence of contrast agents. Background theory relevant to the discussion is summarized in an Appendix. Here, Figure A1 confirms the theoretical relation between the expected performance of a system and the SNR using experimental data from phantoms.

## Methods

Ex vivo T2 mapping was performed on resection specimens from Thai patients with CCA at Khon Kaen University Hospital (KKUH), following the grant of ethics approval by the local Ethics Committee and signature of patient consent forms. Residual specimens were made available for imaging after completion of standard histopathology (formalin fixation, sectioning, staining with haematoxylin and eosin, microscopic examination and confirmatory diagnosis). Tissue sampling and displacement of the MR slice plane both degrade anatomical correlation. Fixation also alters tissue dimensions, elastic properties and MR time constants. Despite this, it was expected that gross morphology and general T2 differences would be preserved.

T2 mapping was carried out with a 3T clinical whole-body system (Achieva, Philips Healthcare, Best, Netherlands) at KKUH, using a multi-echo gradient and spin echo (mGRASE) sequence.^41^ The quadrature body coil (QBC) was used for excitation. Signal reception was carried out using a 16-channel torso phased array coil and a small experimental endoscope coil. SNR was controlled, by varying the number of signal averages (NSA) or the slice thickness (STH). T2 maps were extracted using the vendor’s software, and self-developed Matlab algorithms, fitted to the source images. Both were based on maximum likelihood estimates (MLE), without bias correction but with a threshold to exclude regions of low SNR. Data were archived as screenshots of console displays and as DICOM files, containing the source image and T2 map data. SNR values were found as the local signal average at the first echo time divided by the noise, itself found as the standard deviation of the image in signal-free ROIs divided by the correction factor $\surd \left( {2 - \pi /2} \right)$ between Rician and Gaussian noise.^43^

The endoscope coil was based on an array of magnetically coupled L-C resonators known as a magneto-inductive waveguide.^49^ The complete circuit was fabricated commercially (at Clarydon, Willenhall, UK) by patterning thin copper-clad Kapton to form inductors and parallel plate capacitors. Segmentation and the use of figure-of-eight loops provide passive decoupling from external E fields and B_1_ fields, reducing RF heating and artifacts due to over-excitation. Flexibility allows such coils to be mounted on the shaft of an instrument designed for insertion into the duodenum. Previous experiments using non-magnetic endoscope components and phantoms have shown that such coils do indeed generate few artefacts and can operate with the endoscope shaft curved, while experiments with immersion gel phantoms and fibre optic thermometers have shown little RF heating.^27^

**Figure 1:**
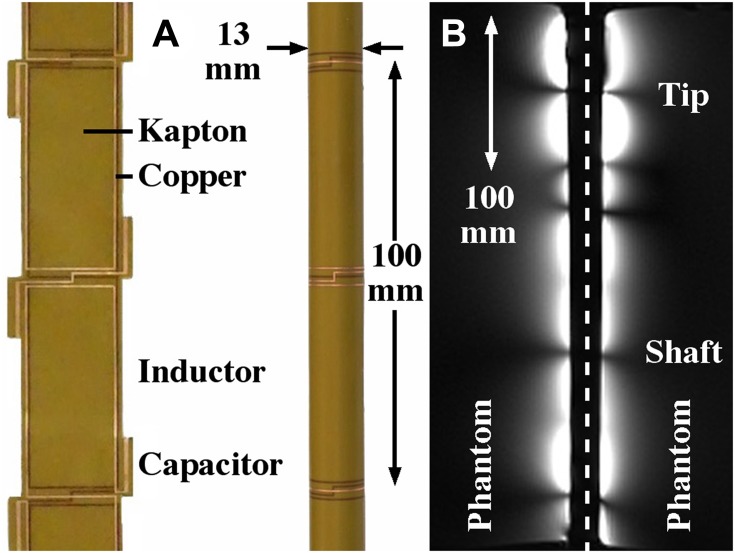
Thin-film circuit, (**A**) before and after mounting on the shaft of a dummy endoscope; (**B**) coronal image of phantoms obtained using the endoscope coil, indicating its reception pattern.

Here, a coil with overall imaging length of 1 m was used, based on a set of nineteen 100 mm long loops formed in 35 μm thick copper on a 25 μm thick Kapton substrate. Each overlapped with neighbouring elements, and the overlap was adjusted at the tip to achieve resonance. The coil was mounted on a cylindrical plastic scaffold with a diameter 2r_0_ = 13 mm using heat-shrink tubing, matched to the system impedance using an inductive tap and connected to an auxiliary coil input via a custom interface (Lambda Z Technologies, Baltimore, USA). Figure 1A shows the thin-film circuit, before and after mounting.

At large distances, the sensitivity of an internal coil based on parallel wires has a $1/{r^2}$ radial dependence.^23^ However, the segmented design used here results in subdivision of the field of view into a set of lobes, with increased sensitivity at the resonant tip.^27^ Figure 1B shows a coronal image obtained with the coil between two cuboid phantoms, which highlights this effect. A peak SNR advantage over a torso coil has been demonstrated to 3r_0_, implying a transverse field of view of around 40 mm and at least a nine-fold advantage near the coil.

Resection specimens were mounted on a cuboid phantom to provide additional signal, and saline bottles were used to mimic body loading. The tip of the endoscope coil was placed on top of the specimen, with the coil axis parallel to the magnet bore, and the arrangement was then surrounded with the torso coil. Images and T2 maps were acquired in the coronal plane rather than the axial plane normally used to allow image correction with a simple radial weighting. In this case, correction is more difficult, but wholly unnecessary for T2 mapping. However, sensitivity might be expected to vary with transverse position *x* approximately as $\left( {r_0^2 + {x^2}} \right){{\rm{}}^{ - 1}}$ immediately beneath the coil tip, implying a three-fold SNR advantage at $x = {r_0}\surd 2$ and equivalent performance at $x = {r_0}\surd 8$.

Verification of system operation and the background theory in the Appendix was also carried out by T2 mapping of phantoms using a similar 3T system. The phantom was a commercial multi-tube test object (TO5 Eurospin II test system, Diagnostic Sonar, Livingston, Scotland)^35^ consisting of a gel-filled perspex holder containing 12 tubes filled with paramagnetically-doped gel. Excitation was performed using the QBC, and signal reception was carried out using the QBC and a 16-channel torso coil; similar results were obtained, but the QBC provided greater consistency due to its uniform FOV.

## Results

**Figure 2:**
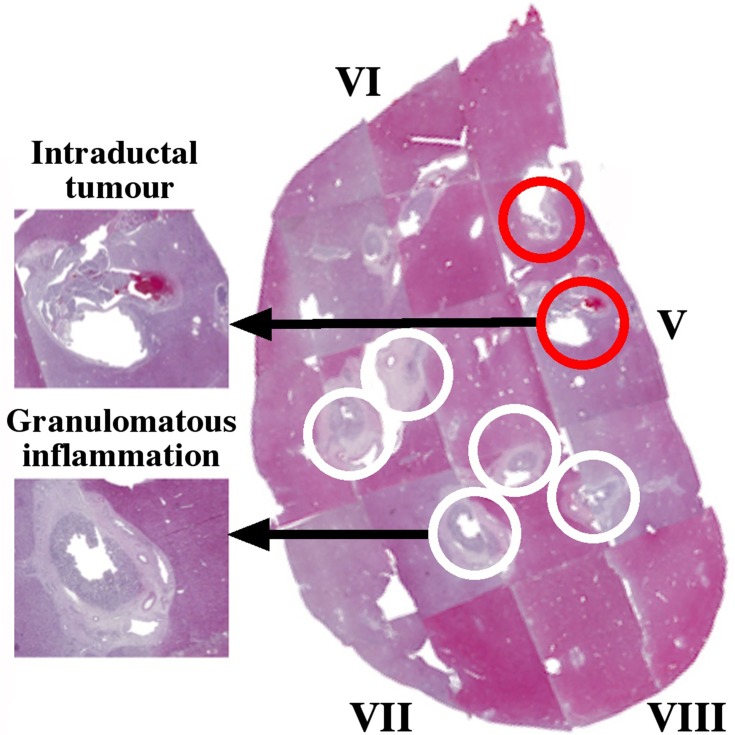
Pathology slide overlay for Specimen 1, with key tissues highlighted. White circles contain granulomatous inflammation; red circles contain intraductal tumours.

Post-operative *ex* vivo human liver specimens with the full range of conditions associated with CCA were examined. The most revealing results were obtained from slices transecting intrahepatic ducts. The first example was a residual slice taken from the extended right hepatectomy of a patient with an intraductal papillary adenoma. Figure 2 shows a reconstruction of the specimen from pathology slide views, in the orientation used for imaging, together with details of key regions. The specimen contained segments V–VIII; white circles enclose portal triads with granulomatous inflammation of vascular lumens surrounded by fibrous connective tissue, while red circles contain intraductal tumours with focal periductal fibrosis.

**Figure 3:**
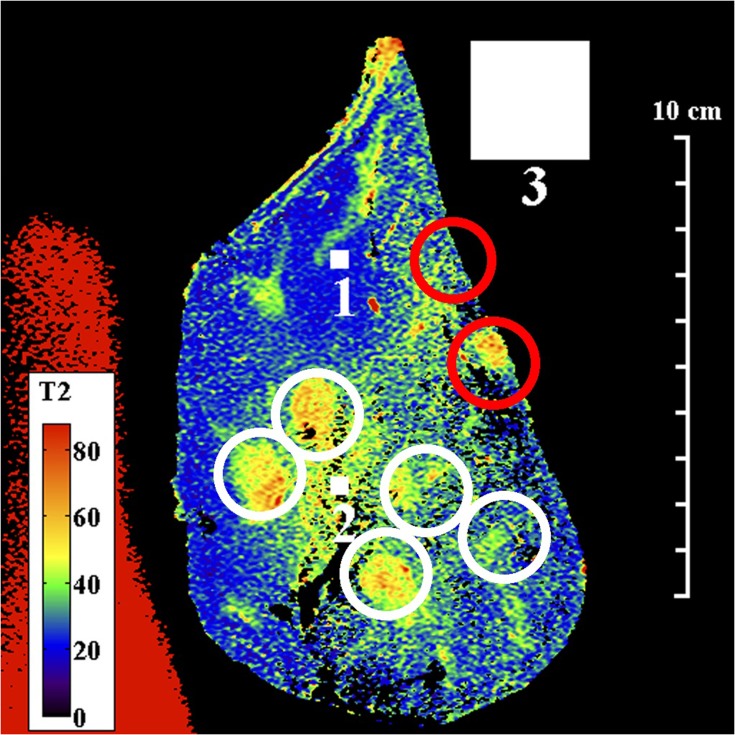
Coronal T2 map of Specimen 1, obtained using the torso coil. White circles contain granulomatous inflammation; red circles contain intraductal tumours. Small and large numbered white squares are regions of interest for signals and noise, respectively.

**Figure 4:**
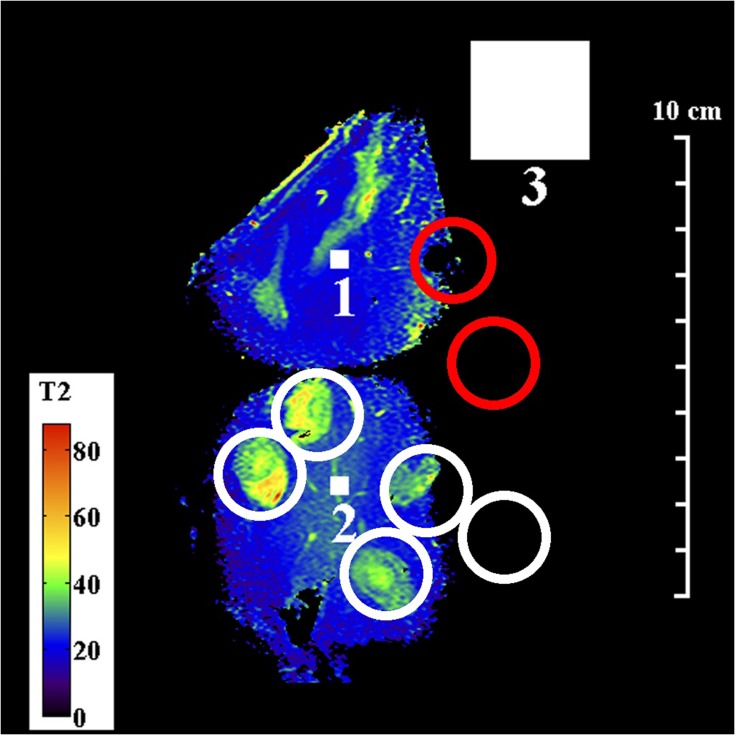
Coronal T2 map of Specimen 1, obtained using the endoscope coil. White circles contain granulomatous inflammation; red circles contain intraductal tumours. Small and large numbered white squares are regions of interest for signals and noise, respectively.

Figures 3 and 4 show T2 maps obtained by matching mono-exponential models to data acquired in a single coronal slice using the torso and endoscope coils, respectively. An mGRASE sequence was used, with a flip angle of 90°, a total of $M = 9$ echoes with a regular echo spacing ${\rm{\Delta }}TE = 15.55ms$, a repetition time $TR = 750ms$, an echo train length $ETL = 45$, a 320 x 200 acquisition matrix, a slice thickness of 2 mm, a reconstruction diameter of 160 mm and 2 signal averages. The small FOV and high resolution are noteworthy. White, numbered square regions of interest (ROIs) are discussed below.

These figures highlight the difference between the two coils. The sensitivity of the external coil was relatively uniform, and yielded a SNR in the range 15–35, allowing the entire specimen to be seen in Figure 3. Regions of granulomatous inflammation (white circles) were identified; however, there were discrepancies in position caused by the use of a sub-surface slice plane. As a result, one intraductal tumour (red circles) now lay at the edge of the capsule while the other appeared absent. Red signal with long T2 at the left-hand side originated from a saline loading bottle. However, although liver parenchyma (blue) was differentiated from ductal tissue (green/yellow/orange), details were blurred and there were significant T2 variations in regions of nominally similar tissue.

In contrast, the sensitivity of the endoscope coil was highly non-uniform, following the sensitivity profile in Figure 1B. Sensitivity was highest beneath the resonant tip, and the SNR now lay in the range 22–440, implying a peak advantage greater than 10. The map in Figure 4 was segmented vertically by the coil construction into 50 mm long sections, and restricted horizontally by the radial dependence of sensitivity to around 40 mm. Due to the SNR cutoff, only part of the specimen lay within the effective FOV, so that only four regions of granulomatous inflammation could be clearly identified. Despite this, liver parenchyma was found to have a uniform, short T2 (ca 20 ms), while the multiple tissue layers involved in granulomatous reaction were now distinct; all had closely-spaced T2 values, in the range 40–50 ms. This structure may contribute to the appearance of some ducts during conventional imaging.

**Figure 5:**
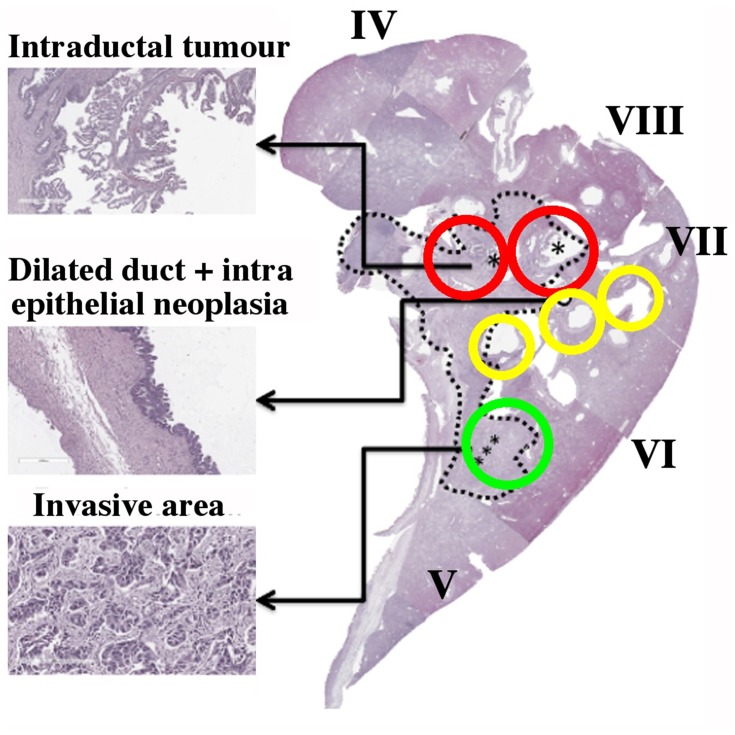
Pathology slide overlay for Specimen 2, with key tissues highlighted. The green circle contains part of the large invasive area (dashed line), red circles contain intraductal tumours and the yellow circles contain intraepithelial neoplasia at the edge of dilated ducts. Asterisks indicate sites with similar pathology, investigated in detail.

Other specimens offered less advantageous slice planes. Figure 5 shows the pathology slide overlay from a second example, again from an extended right hepatectomy. This specimen consisted of segments IV–VIII, and contained intraductal tumours in the hilar region (red circles), together with a much larger invasive area enclosed by the dashed line where cancer had advanced beyond the duct wall into the liver parenchyma. The invasive area contained varying densities of tumour cells, desmoplastic stroma and necrosis (for example, as shown in the sub-region circled green). Microscopic examination also showed intraepithelial neoplasia in adjacent dilated intrahepatic ducts (yellow circles).

**Figure 6:**
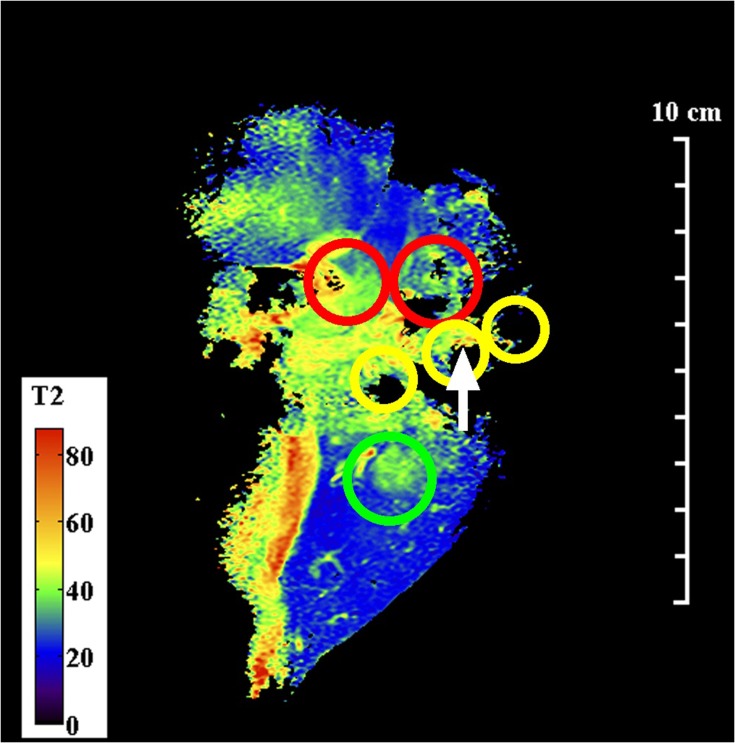
Coronal T2 map of Specimen 2, obtained using the endoscope coil. The green circle contains part of the invasive area, red circles contain intraductal tumours and the yellow circles contain intraepithelial neoplasia at the edge of dilated ducts. The arrow indicates an area with long T2, positively correlated with neoplasia.

Figure 6 shows the coronal T2 map obtained with the endoscope coil. Here, the segmentation introduced by the coil design was less severe, but partial volume effects were expected in hilar ducts arising from the 2 mm slice thickness. Despite this, the biliary tree and dilated ducts were visualized, invasive areas were readily located and intraductal tumours were identified as heterogeneous tissue with long T2 (60–70 ms). Very high resolution is needed to distinguish intraepithelial neoplasia, which might also be expected to have long T2; a one positive correlation (arrowed) is shown at the edge of a dilated duct.

**Figure 7:**
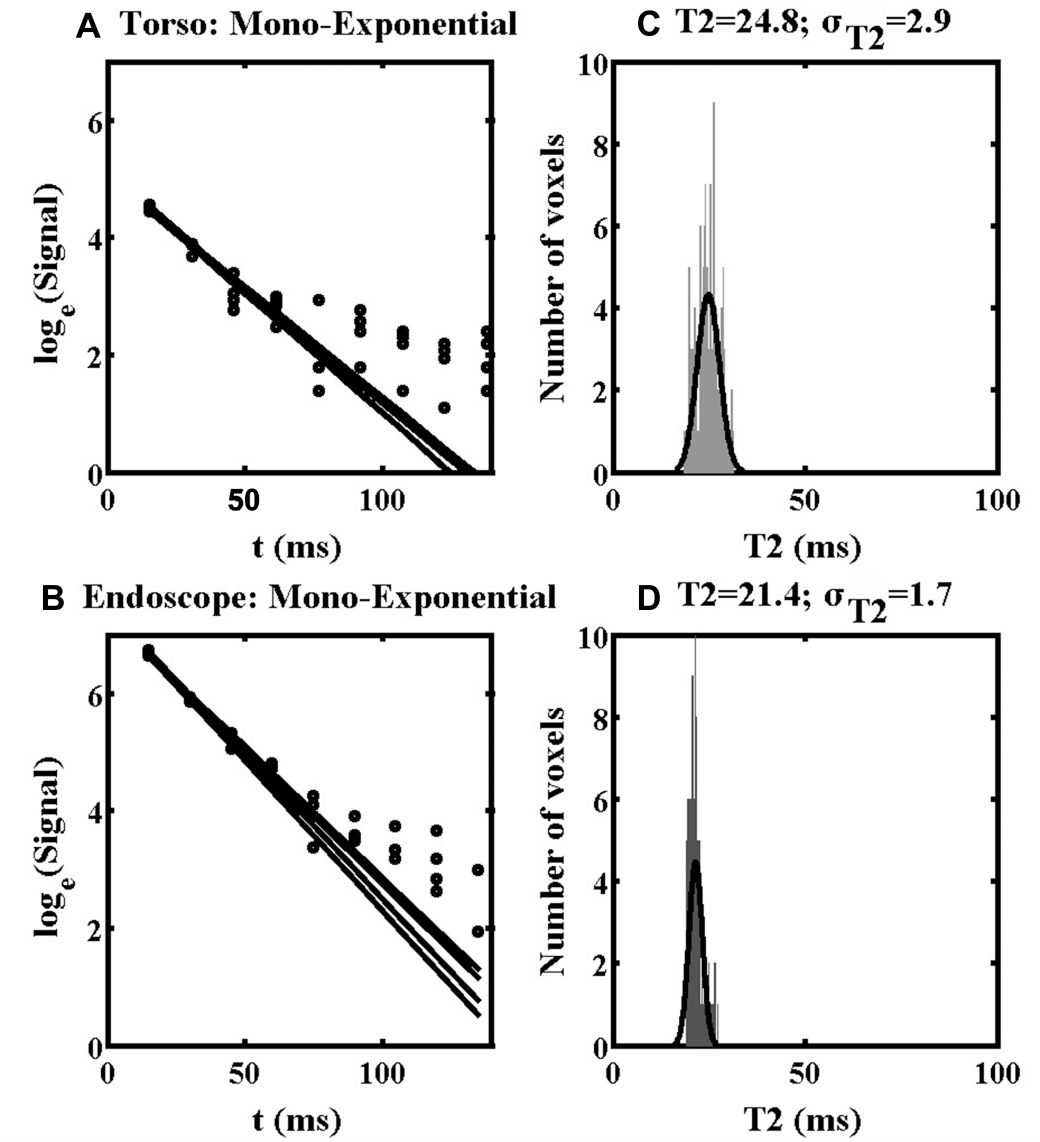
Mono-exponential fitting lines (**A, B**) and distributions of T2 values (**C, D**) obtained using the torso coil (upper) and endoscope coil (lower) in ROI 1 of Specimen 1.

Other aspects of parameter estimation were considered, including the effects of SNR, tissue heterogeneity and model order, by detailed investigation of the 11x11 regions of interest (ROI) in specimen 1, numbered 1 and 2 in Figures 3 and 4. The larger ROI numbered 3 was used for noise measurement. Figure 7 shows results obtained from ROI 1, which corresponds to homogeneous liver parenchyma. Figure 7A and B show a reduced number of fitting lines obtained by matching mono-exponential models to data obtained using the torso and endoscope coils, respectively. The data are on a logarithmic scale and the increased signal obtained using the endoscope coil is clear. Figure 7C and D show the T2 distributions extracted in each case. The low SNR of the torso coil resulted in an unrealistic estimate of the mean of T2 (which is artificially increased by fitting to noise), accompanied by a large standard deviation. The higher SNR of the endoscope coil resulted in a lower T2 and a reduced value of σ_T2N_. It is this reduction that is responsible for clarifying anatomical features in Figure 4. However, the T2 value (21.4 ms) is substantially lower than literature values for liver parenchyma in vivo (31–34 ms), presumably due to formalin fixation.^42^

**Figure 8:**
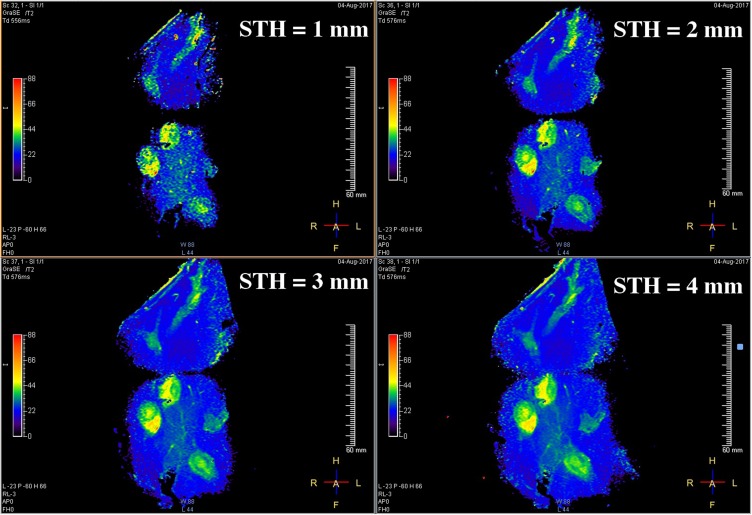
Coronal T2 maps of Specimen 1 obtained using the endoscope coil with different slice thicknesses between 1 mm and 4 mm.

As is well known, SNR may also be increased using a larger slice thickness or number of averages, at a price of increased partial volume effects or acquisition time. One effect is to improve feature definition; another is to increase the FOV allowed by the SNR cut-off. Figure 8 shows T2 maps of specimen 1 obtained using the endoscope coil with $STH = 1 \ldots 4mm$ which illustrate the latter effect. For highly homogeneous tissue, the standard deviation of T2 due to noise alone (σ_T2N_) should be directly proportional to $SN{R^{ - 1}}$(see Equation A8 in the Appendix). Since ${\sigma _{T2N}} = kST{H^{ - 1}}$ is directly proportional to slice thickness, a linear relation of the form ${\sigma _{T2N}} = kST{H^{ - 1}}$ would then be expected.

**Figure 9:**
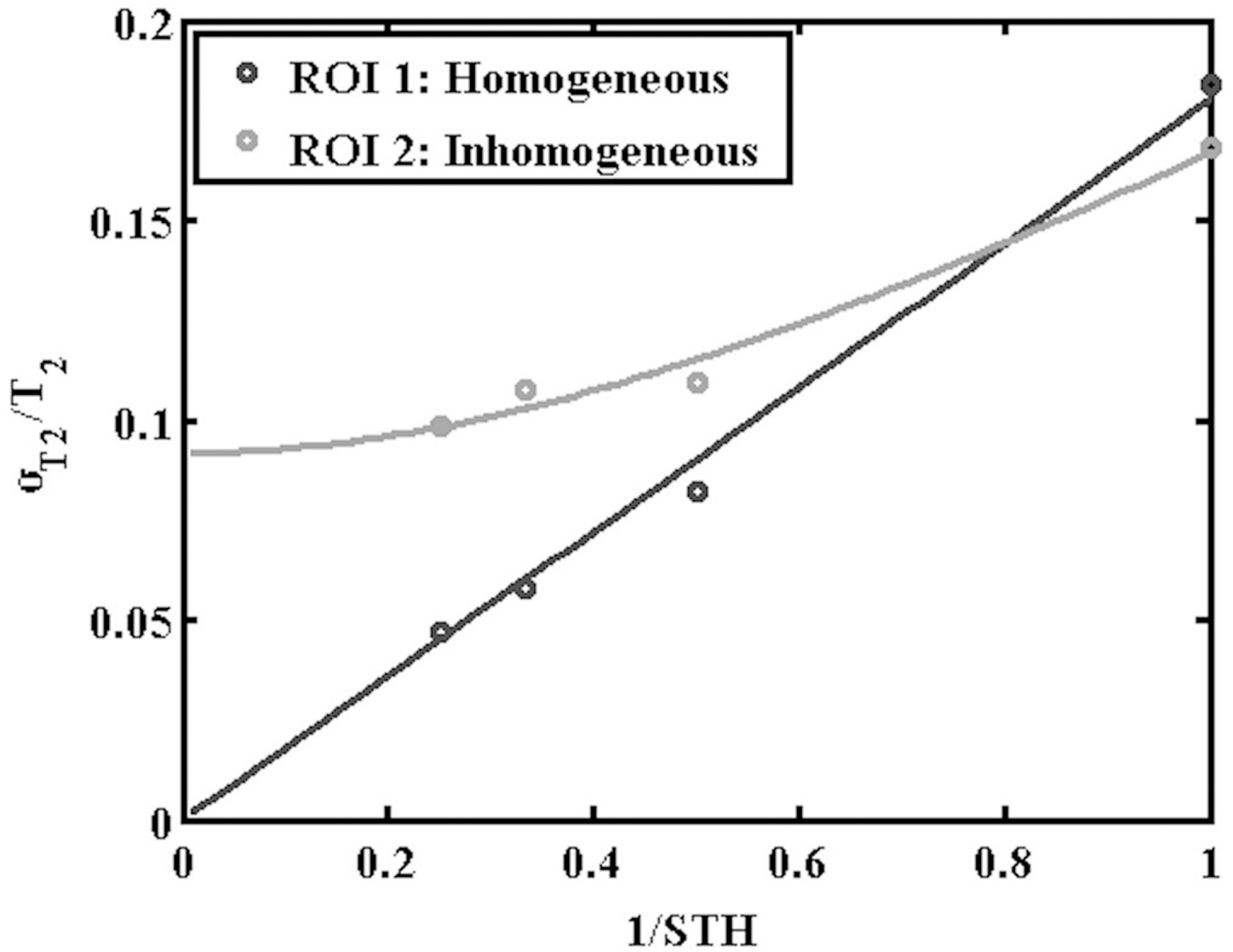
Comparison of extracted variations of normalised standard deviation of T2 with the inverse of the slice thickness with fitting lines, for the approximately homogeneous tissue (black line) and clearly heterogeneous tissue in ROIs 1 and 2 of Specimen 1 (grey line).

This trend was verified by matching to data obtained from homogeneous liver parenchyma in ROI 1 using the endoscope coil, as shown by the black line in Figure 9; there is clearly good agreement. Increasing SNR cannot, however, compensate for tissue variability. Assuming that heterogeneity may be described in terms of a Gaussian distribution of T2 values with a standard distribution σ_T2H_, the overall variation *σ_T2_* must contain contributions from both σ_T2N_ and σ_T2H_ (Equation A9). This relation was verified for ROI 2, which clearly contains heterogeneous tissue, by matching data to the relation ${\sigma _{T2}} = \surd \left\{ {\sigma _{T2H}^2 + {{\left( {kST{H^{ - 1}}} \right)}^2}} \right\}$ as shown by the grey line in Figure 9. Once again, good agreement is obtained.

**Figure 10:**
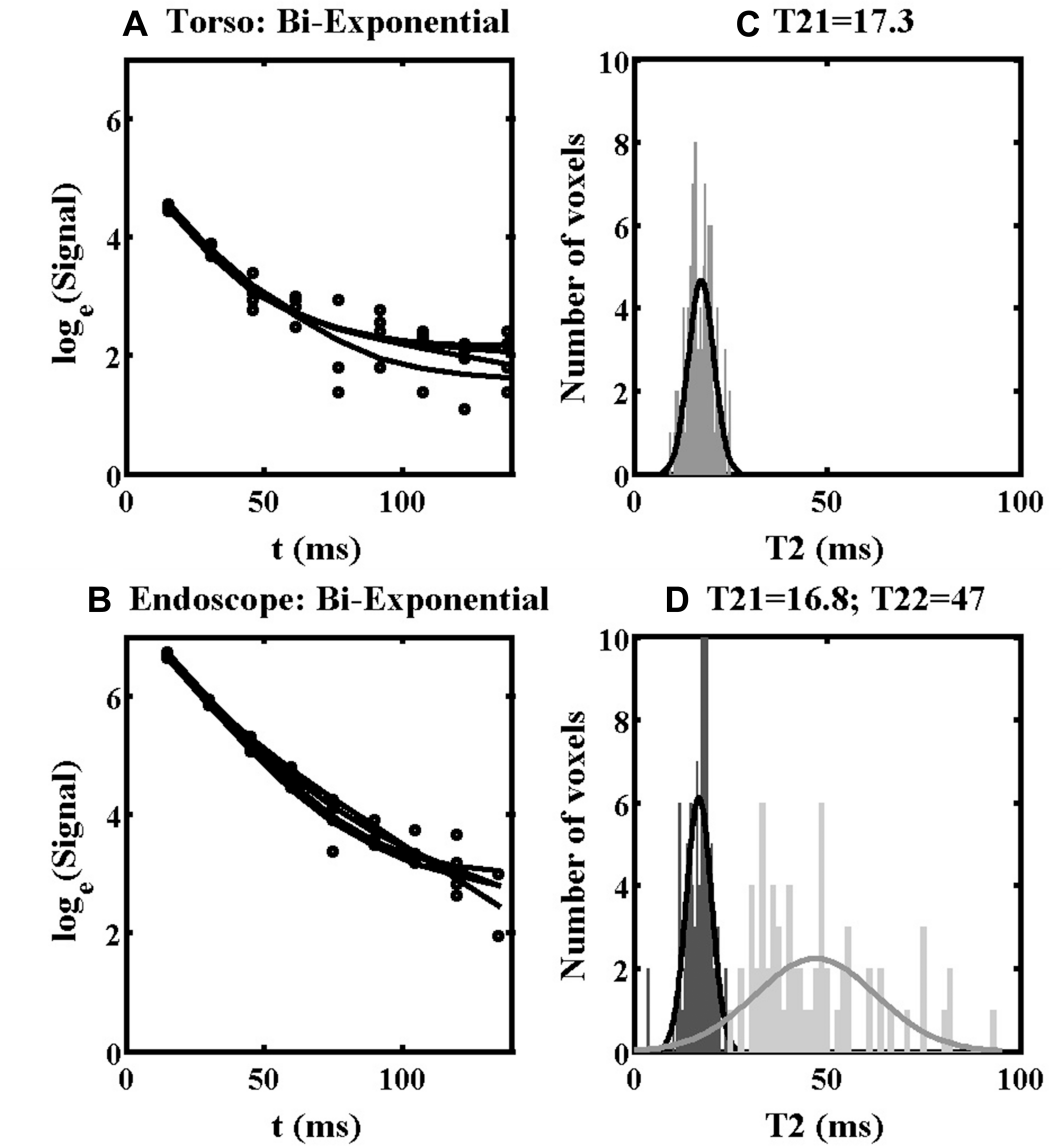
Bi-exponential fitting lines (**A, B**) and distributions of T21 and T22 values (**C, D**) obtained using the torso coil (upper) and endoscope coil (lower) in ROI 1 of Specimen 1.

The increased SNR offered by the endoscope coil should, in addition, improve testing of higher-order relaxation models. Figure 10 shows the results obtained by matching bi-exponential models to data from ROI 1. Figures 10A and B show fitting lines obtained using the torso and endoscope coils, respectively, and Figure 10C and D show the corresponding distributions of estimated time constants. For the torso coil, only the short time constant T21 is shown, as the SNR was insufficient to yield a recognizable distribution for the long time constant T22. For the endoscope coil, distributions were extracted for both T21 and T22. Both have large standard deviations, as predicted by the discussion of Equation A10. However, these results suggest that the mono-exponential estimate of T2 was only slightly overestimated, since the component with longer T22 (50 ms) is relatively weak. The results above confirmed that increasing SNR improves the accuracy and reduces the spread of T2 values estimated using low-order models, and increases the ability to achieve realistic parameters for higher-order models.

## Discussion

This study has shown by comparison with an external coil that improved tissue differentiation may be obtained in *ex viv*o T2 mapping of cholangiocarcinoma resection specimens using an endoscope coil designed ultimately for internal use. Experimental demonstration was performed using a clinical MRI scanner at 3T, operating commercial mGRASE sequences and T2 mapping software. Results have been correlated with histopathology. The layered structure of granulomatous inflammation – an early disease indicator – has been revealed, together with other signs of early- and late-stage disease.

Locally raising SNR using an internal coil allows the performance of a clinical scanner to be raised closer to that of an NMR spectrometer, without the disadvantages of a larger slice thickness (which leads to increased partial volume effects) or number of averages (which results in increased scanning time and RF heating). The use of relaxometry rather than imaging allows this advantage to be exploited directly, despite the spatial sensitivity variation inherent in any reduction of body noise by a smaller field-of-view. Software correction of image brightness and the fiducial markers used to locate the correction centre are both unnecessary. Restrictions on coil orientation are relaxed and the slice orientation may be arbitrary, major advantages for curvilinear lumens. Limited interaction between internal coils of the type demonstrated and an external array should allow the fusion of local, high-resolution T2 mapping with full torso imaging, enabling rapid identification and optimization of the FOV. While no compensation can be made for tissue heterogeneity, the reduction of parameter spread due to thermal noise improves the differentiation of similar T2 values, and renders fitting to higher-order relaxation models more viable. This use of internal coils may have implications for difficult imaging problems, such as delineating CCA precursors that lack the distinguishable perfusion patterns exploited in dynamic contrast enhanced MRI.

Translation to in vivo use involves significant further work. The endoscopic coil demonstrated here is designed to avoid coupling to the scanner during excitation. However, further safety testing is required, following the incorporation of optical imaging and steering. A procedure similar to endoscopic retrograde cholangiopancreatography must then be developed to place the coil close to the target tissue. The coil demonstrated here has axial and radial fields-of-view of around 10 cm and 2 cm, sufficient to encompass the ampulla of Vater and some extrahepatic ducts. Investigation of intrahepatic ducts would require cannulation using a smaller internal coil (for example, a magneto-inductive catheter receiver, which has already demonstrated in guide-wire compatible form^50^). SNR advantage must then be demonstrated despite patient motion, for example, using respiratory gating, anticholinergic drugs or motion-insensitive sequences. Finally, T2 values require investigation, to establish typical parameter values and identify multi-exponential processes. Procedural complexity and patient risk suggest that internal coils are unlikely to be used for screening, but may find uses where diagnosis of early-stage disease is unclear or margins must be accurately identified to reduce recurrence. However, the scale of the disease problem in southeast Asia and lack of alternative solutions suggest that further investigations are justified.

## References

[1] Khan S, Thomas HC, Davidson BR, Taylor-Robinson SD. Cholangiocarcinoma. *Lancet*. 2005;366(9493):1303–1315. doi:10.1016/S0140-6736(05)67530-7 16214602

[2] Patel T. Increasing incidence and mortality of primary intrahepatic cholangiocarcinoma in the United States. *Hepatology*. 2001;33(6):1353–1357. doi:10.1053/jhep.2001.25087 11391522

[3] Taylor-Robinson SD, Toledano MB, Arora S, et al. Increase in mortality rates from intrahepatic cholangiocarcinoma in England and Wales 1968-1998. *GUT*. 2001;48(6):816–820. doi:10.1136/gut.48.6.816 11358902 PMC1728314

[4] Sripa B, Bethony JM, Sithithaworn P, et al. Opisthorchiasis and *Opisthorchis*-associated cholangiocarcinoma in Thailand and Laos. *Acta Trop*. 2011;120(Suppl 1):S158–S168. doi:10.1016/j.actatropica.2010.07.006 20655862 PMC3010517

[5] Sripa B, Brindley PJ, Mulvenna J, et al. The tumorigenic liver fluke Opisthorchis viverrini – multiple pathways to cancer. *Trends Parasitol*. 2012;28(10):395–407. doi:10.1016/j.pt.2012.07.006 22947297 PMC3682777

[6] Kuntikeo N, Loilome W, Thinkhamrop B, Chamadol N, Yongvanit P. Framework and strategy to effectively combat cholangiocarcinoma in Thailand. *PLoS Negl Trop Dis*. 2016;10(1):e0004293. doi:10.1371/journal.pntd.0004293 26797527 PMC4721916

[7] Bismuth H, Corlette MB. Intrahepatic cholangioenteric anastomosis in carcinoma of the hilus of the liver. *Surg Gyn Obs*. 1975;140:170–178. 1079096

[8] Lim JH. Cholangiocarcinoma: morphologic classification according to growth pattern and imaging findings. *Am J Roentgenol*. 2003;181(3):819–827. doi:10.2214/ajr.181.3.1810819 12933488

[9] Nakanuma Y, Curado MP, Franceschi S, Gores G, Paradis V, Sripa B. Intrahepatic cholangiocarcinoma In: Bosman FT, Hruba RH, Theise ND, editors. *WHO Classification of Tumors of the Digestive System*. Lyon: IARC Press; 2010:217–224.

[10] Komuta M, Govaere O, Vandecaveye V, et al. Histological diversity in cholangiocarcinoma reflects the different cholangiocyte phenotypes. *Hepatology*. 2012;55(6):1876–1888. doi:10.1002/hep.25595 22271564

[11] Nakanuma Y, Kakuda Y. Pathologic classification of cholangiocarcinoma: new concepts. *Best Pract Res Clin Gastroent*. 2015;29(2):277–293. doi:10.1016/j.bpg.2015.02.006 25966428

[12] Wongkham S, Silsirivanit A. State of serum markers for detection of cholangiocarcinoma. *Asian Pacific J Cancer Prev*. 2012;13(Suppl):17–27. 23480761

[13] Chamadol N, Pairojkul C, Khuntikeo N. Histological confirmation of periductal fibrosis from ultrasound diagnosis in cholangiocarcinoma patients. *J Hepatobil Pancreat Sci*. 2014;21(5):316–322. doi:10.1002/jhbp.2014.21.issue-5 24420706

[14] Crane CH, Macdonald KO, Vauthey JN, et al. Limitations of conventional doses of chemoradiation for unresectable biliary cancer. *Int J Radiat Oncol Biol Phys*. 2002;53(4):969–974. doi:10.1016/S0360-3016(02)02845-6 12095564

[15] Khuntikeo N, Pugkhem PA, Titapun A, Bhudhisawasdi V. Surgical management of perihilar cholangiocarcinoma: a Khon Kaen experience. *J Hepatobil Pancreat Sci*. 2014;21(8):521–524. doi:10.1002/jhbp.2014.21.issue-8 24464976

[16] Hennedige TP, Neo WT, Venkatesh SK. Imaging of malignancies of the biliary tract – an update. *Cancer Imaging*. 2014;14:14–21. doi:10.1186/1470-7330-14-14 25608662 PMC4331820

[17] Lee DH, Lee JM, Kim KW, et al. MR imaging findings of early bile duct cancer. *J Magn Reson Imag*. 2008;28(6):1466–1475. doi:10.1002/jmri.v28:6 19025934

[18] Kim JY, Lee JM, Han JK, et al. Contrast-enhanced MRI combined with MR cholangiopancreatography for the evaluation of patients with biliary structures: differentiation of malignant from benign bile duct strictures. *J Magn Reson Imag*. 2007;26(2):304–312. doi:10.1002/(ISSN)1522-2586 17623893

[19] Kim H, Lim JH, Jang KT, et al. Morphology of intraductal papillary neoplasm of the bile ducts: radiologic-pathologic correlation. *Abdom Imag*. 2011;36(4):438–446. doi:10.1007/s00261-010-9636-2 20623279

[20] Joo I, Lee JM. Imaging bile duct tumours: pathologic concepts, classification, and early tumour detection. *Abdom Imag*. 2013;38:1334–1350. doi:10.1007/s00261-013-0027-3 23925840

[21] Eryaman Y, Oner Y, Atalar E. Design of internal coils using ultimate intrinsic SNR. *Magn Reson Mater Phys*. 2009;22(4):221–228. doi:10.1007/s10334-009-0167-1 19326160

[22] Kantor HL, Briggs RW, Balaban RS. In vivo ^31^P nuclear magnetic resonance measurements in canine heart using a catheter-coil. *Circ Res*. 1984;55(2):261–266. doi:10.1161/01.RES.55.2.261 6744535

[23] Atalar E, Bottomley PA, Ocali O, et al. High resolution intravascular MRI and MRS by using a catheter receiver coil. *Mag Res Med*. 1996;36(4):596–605. doi:10.1002/(ISSN)1522-2594 8892213

[24] Inui K, Nakazawa S, Yoshino J, et al. Endoscopic MRI: preliminary results of a new technique for visualization and staging of gastrointestinal tumors. *Endoscopy*. 1995;27(07):480–485. doi:10.1055/s-2007-1005752 8565886

[25] Ladd ME, Quick HH. Reduction of resonant RF heating in intravascular catheters using coaxial chokes. *Magn Reson Med*. 2000;43(4):615–619. doi:10.1002/(ISSN)1522-2594 10748440

[26] Weiss CR, Georgiades C, Hofmann LV, et al. Intrabiliary MR imaging: assessment of biliary obstruction with the use of an intraluminal MR receiver coil. *J Vasc Interv Radiol*. 2006;17(5):845–853. doi:10.1016/S1051-0443(07)60823-9 16687751

[27] Syms RRA, Kardoulaki E, Rea M, et al. Magneto-inductive magnetic resonance imaging duodenoscope. *PIER*. 2017;159:125–138. doi:10.2528/PIER17062104

[28] Poon CS, Henkelman RM. Practical T2 quantitation for clinical applications. *J Magn Reson Imag*. 1992;2(5):541–553. doi:10.1002/(ISSN)1522-2586 1392247

[29] Bernardino ME, Small W, Goldstein J, et al. Multiple NMR T2 relaxation values in human liver tissue. *Am J Roentgenol*. 1983;141(6):1203–1208. doi:10.2214/ajr.141.6.1203 6606317

[30] Bottomley PA, Foster TH, Argersinger RE, Pfeifer LM. A review of normal tissue hydrogen NMR relaxation times and relaxation mechanism from 1-100 MHz: dependence on tissue type, NMR frequency, temperature, species, excision, and age. *Med Phys*. 1984;11(4):425–448. doi:10.1118/1.595535 6482839

[31] Kamman RL, Go KG, Stomp GP, Hulstaert CE, Berendsen HJ. Changes of relaxation times T_1_ and T_2_ in rat tissues after biopsy and fixation. *Magn Reson Imaging*. 1985;3(3):245–250. doi:10.1016/0730-725X(85)90353-4 3908869

[32] Carr HY, Purcell EM. Effects of diffusion on free precession in nuclear magnetic resonance experiments. *Phys Rev*. 1954;94(3):630–638. doi:10.1103/PhysRev.94.630

[33] Majumdar S, Orphanoudakis SC, Gmitro A, O’Donnell M, Gore JC. Errors in the measurement of T_2_ using multiple echo MRI techniques. II. Effects of static field inhomogeneity. *Magn Reson Med*. 1986;3:562–574. doi:10.1002/mrm.1910030410 3747818

[34] Kjos BO, Ehman RL, Brant-Zawadzki M. Reproducibility of T_1_ and T_2_ relaxation times calculated from routine MR imaging sequences: phantom study. *AJR*. 1985;144:277–283. 10.2214/ajr.144.6.1157 2988317

[35] Lerski RA, de Certaines JH. Performance assessment and quality control in MRI by Eurospin test objects and protocols. *Magn Reson Imag*. 1993;11(6):817–833. doi:10.1016/0730-725X(93)90199-N 8371637

[36] Bottomley PA, Hardy CJ, Argersinger RE, Allen-Moore G. A review of ^1^H nuclear magnetic resonance relaxation in pathology: are T_1_ and T_2_ diagnostic? *Med Phys*. 1987;14:1–37. 3031439 10.1118/1.596111

[37] Meiboom S, Gill D. Modified spin-echo method for measuring nuclear relaxation times. *Rev Sci Inst*. 1958;29(8):688–691. doi:10.1063/1.1716296

[38] Deichmann R, Adolf H, Kuchenbrod E, Noth U, Schwarzbauer C, Haase A. Compensation of diffusion effects in T2 measurements. *Magn Reson Med*. 1995;33(1):113–115. doi:10.1002/(ISSN)1522-2594 7891524

[39] Mylnarik V, Degrassi A, Toffanin R, Jahr O, Vittur F. A method for generating magnetic resonance microimaging T2 maps with low sensitivity to diffusion. *Magn Reson Med*. 1996;35(3):423–425. doi:10.1002/mrm.v35.3 8699955

[40] Sussman MS, Vidarsson L, Pauly JM, Cheng HL. A technique for rapid single-echo spin-echo T_2_ mapping. *Magn Reson Med*. 2010;64(2):536–545. doi:10.1002/mrm.22454 20665797

[41] Baessler B, Schaarschmidt F, Stehning C, Schnackenburg B, Maintz D, Bunck AC. Cardiac T_2_-mapping using a fast gradient echo spin echo sequence - first in vitro and in vitro experience. *J Cardiovasc Magn Reson*. 2015;17(1):67. doi:10.1186/s12968-015-0177-2 26231927 PMC4522069

[42] Bojorquez J, Bricq S, Acquitter C, Brunotte F, Walker PM, Lalande A. What are normal relaxation times of tissue at 3T? *Magn Reson Imag*. 2017;35:69–80. doi:10.1016/j.mri.2016.08.021 27594531

[43] Gudbjartsson H, Patz S. The Rician distribution of noisy MRI data. *Magn Reson Med*. 1995;34:910–914. doi:10.1002/(ISSN)1522-2594 8598820 PMC2254141

[44] Sijbers J, den Dekker AJ, Raman E, van Dyck D. Parameter estimation from magnitude MR images. *Int J Imag Syst Technol*. 1999;10(2):109–114. doi:10.1002/(ISSN)1098-1098

[45] Weiss GH, Ferretti JA. Optimal design of relaxation time experiments. *Prog NMR Spectrosc*. 1988;20(4):317–335. doi:10.1016/0079-6565(88)80009-8

[46] Bjarnason TA, McCreary CR, Dunn JF, Mitchell JR. Quantitative T_2_ analysis: the effects of noise, regularization, and multivoxel approaches. *Magn Reson Imag*. 2010;63:212–217. 10.1002/mrm.22173 20027599

[47] Clayden NJ, Hesler BD. Multiexponential analysis of relaxation decays. *J Magn Reson*. 1992;98:271–282.

[48] Graham SJ, Stanchev PL, Bronskill MJ. Criteria for analysis of multicomponent tissue T_2_ relaxation data. *Magn Reson Med*. 1996;35(3):370–378. doi:10.1002/mrm.v35.3 8699949

[49] Shamonina E, Kalinin VA, Ringhofer KH, Solymar L. Magneto-inductive waveguide. *Electron Lett*. 2002;38(8):371–373. doi:10.1049/el:20020258

[50] Syms RRA, Young IR, Ahmad MM, Taylor-Robinson SD, Rea M. Magneto-inductive catheter receiver for magnetic resonance imaging. *IEE Trans Biomed Eng*. 2013;60(9):2421–2431. doi:10.1109/TBME.2013.2258020 23591471

